# Evaluating the effectiveness of the Family Connections program for caregivers of youth with mental health challenges, part I: A quantitative analysis

**DOI:** 10.1111/hex.13205

**Published:** 2021-02-13

**Authors:** Tali Z. Boritz, Natasha Y. Sheikhan, Lisa D. Hawke, Shelley F. McMain, Joanna Henderson

**Affiliations:** ^1^ Centre for Addiction and Mental Health Toronto Ontario Canada; ^2^ Department of Psychiatry University of Toronto Toronto Ontario Canada; ^3^ Dalla Lana School of Public Health University of Toronto Toronto Ontario Canada

**Keywords:** adolescent, caregivers, dialectical behavior therapy, family relations, mental health, peer group

## Abstract

**Introduction:**

Caregivers of youth with mental health (MH) challenges are often faced with complex problems in relation to caring for their youth. Family Connections™ (FC) is a 12‐week skills training program for families of individuals with MH challenges, developed originally for Borderline Personality Disorder. Research is needed to examine the effectiveness of FC for caregivers of youth with diverse MH challenges.

**Objective:**

To examine the effectiveness of FC for caregivers of youth with MH challenges.

**Methods:**

A total of 94 caregivers of youth with MH challenges participated in FC, across three sites in Ontario, Canada. Assessments occurred at baseline, 6 weeks, 12 weeks and follow‐up. Primary outcomes include the Burden Assessment Scale and The Stress Index for Parents of Adolescents. Secondary outcomes included the caregiver's report of child behaviour, affect, mastery, coping and grief. Linear mixed model analyses were conducted, where time and the time × site interaction were defined as the fixed effects.

**Results:**

Statistically significant improvements over time were observed across outcome measures, including caregiver burden, grief, coping, and other measures. The time × site interaction was only significant for burden (*P* = .005).

**Conclusion:**

This study demonstrates the effectiveness of FC for caregivers of youth with MH challenges. Future research should focus on differences across geographical sites and facilitation models.

**Patient or public contribution:**

Caregivers were involved in the facilitation of FC. A person with lived experience was involved in analysing the data, reporting the results, and drafting the manuscript.

## 1 INTRODUCTION

Youth aged 15 to 29 represent 19.2% of the Canadian population.^1^ On a global scale, the leading causes of disability among youth are mental health (MH) and substance use related disorders.^2^ In a Canadian context, suicide is the second leading cause of death among youth.^3^ Often, mental illness initially presents during childhood or adolescence; this is especially the case for anxiety disorders and impulse control disorders.^4^ Poorer mental health among youth is related to a variety of individual and interpersonal issues, such as stigma, substance use and lower educational achievements.^5^, ^6^

The difficulties experienced by youth with MH challenges often extend to their families.^7^ Family members have been shown to experience high levels of distress, confusion and fear regarding their knowledge about their youth's MH challenge.^8^ Physical, social and financial struggles of family members of youth with MH challenges have also been reported in the literature, including barriers to formal care, changes to service delivery, stigma and a lack of availability of services.^9^, ^10^, ^11^, ^12^

Family and caregivers of youth with MH challenges may lack knowledge regarding their youth's disorders and may not have adequate coping strategies to manage their caregiving role.^13^ They frequently report disproportionate rates of caregiver burden, lower mastery and heightened distress compared to the general population.^7^, ^14^ In some cases, family members report feeling pressured to give up other roles and activities to care for their youth ^15^ and may experience rejection or blame from extended family members, friends and community members who misunderstand or disapprove of the youth's behaviour and needs.^16^ Accordingly, it is important to consider ways to support families, as well as their youth.

### 1.1 Interventions for families of youth with MH challenges

Involving family members in youth MH services is of particular importance since youth often live with one or both parents and are subject to their authority and support. When family members are involved in services, not only is the youth's recovery facilitated, but the well‐being of family members is heightened.^17^ Family involvement has been shown to be associated with improved parent engagement, including increased motivation, improved expectations and reduced perceived barriers to treatment.^18^ Among family members, family involvement is also shown to decrease self‐reported experiences of mental distress, build coping skills and empower family members.^19^, ^20^, ^21^, ^22^ However, there can be a number of barriers associated with family involvement in treatment. For example, Baker‐Ericzén, Jenkins and Haine‐Schlagel^23^ found that that family members report inadequate support from the service system, feeling unsupported by therapist and feeling overwhelmed by the complexities of the needs of the youth and the family.

Several interventions for family members are clinician‐led and focus on family change by addressing parenting practices, the family environment and problem‐solving, while taking into account the youth's psychosocial environment.^24^, ^25^ More recently, peer‐led interventions led by individuals with share lived experiences can also provide education and support for family members.^26^, ^27^, ^28^ Peer‐led interventions are a cost‐effective alternative to clinician‐led inventions, while creating greater trust and rapport among participants.^28^ Current peer‐led interventions have shown promising results, such as reducing burden, improving empowerment, hope and self‐esteem^29^, ^30^, ^31^; however, there is a dearth of literature that focus on families of youth with mental health problems.

*Peer facilitated interventions, which are less* resource‐intensive, may be good candidates for scale up as they have the strong impact of peer‐run programs (e.g. empowerment) and are cost‐effective.^32^ However, further work is needed to develop and scale up peer‐led services designed to support family members by teaching coping skills to help them manage the events that result from having a youth with MH challenges.

### 1.2 The Family Connections™ program

Family Connections™ (FC) is a manualized skill‐based program that was originally created for family members with a relative with borderline personality disorder (BPD) and widely delivered by peer facilitators to family members.^15^ FC aims to support family members as they attempt to effectively support their loved one, while simultaneously enhancing their own well‐being.^15^ This 12‐week group‐based intervention has been implemented in community settings and focuses on the provision of information and research on mental health and family functioning, coping skills, family skills and social support.^15^

FC was created based on two theoretical models. The stress‐coping‐and‐adaptation (SCA) model approach by Lazarus and Folkman^33^ focuses on the strengths, resources, and adaptive capacities that individuals draw upon when their functioning is disrupted by major life events and challenges. Coping strategies are thought to act as the mediator in managing the stressors that result from the impact of mental illness on the family environment.^33^ The second theoretical model is the Dialectical Behaviour Therapy (DBT) model, a cognitive‐behavioural treatment approach that has demonstrated effectiveness in treating BPD and other psychological problems.^34^ FC draws from DBT to provide family members with a set of coping skills that facilitate a balanced view of their needs and the needs of their loved one with mental health challenges.^35^

In the initial study of FC by Hoffman, Fruzzetti, Buteau, Neiditch, Penney, Bruce, Hellman and Struening,^36^ 44 participants in the program reported significant decreases in burden (i.e. stressors due to relative's symptomology) and grief (i.e., cognitive and psychological problems associated with having a relative with mental illness), as well as a significant increase in mastery (i.e., self‐management skills to cope with having a relative with mental illness) from baseline to a three month post‐baseline. In a replication study by Hoffman, Fruzzetti and Buteau,^15^ 55 FC participants were assessed at pre‐, post‐ and 3 month post‐program intervals and were found to once again show significant improvements on all well‐being variables. Male and female participants reported similar improvements at program completion, although females remained higher on the grief scale as compared to males despite significant improvements for both sexes. Rajalin, Wickholm‐Pethrus, Hursti and Jokinen^37^ conducted a pilot study evaluating FC, specifically for family members of individuals with suicidal behaviour. This study found a significant reduction of the sense of burden and an improvement in the well‐being and psychic health of the family member.

More recently, Flynn, Kells, Joyce, Corcoran, Herley, Suarez, Cotter, Hurley, Weihrauch and Groeger^38^ examined the effectiveness of FC (n = 51) versus an optimized treatment‐as usual (OTAU; n = 29) for families of individuals with BPD. Reflecting the aforementioned studies, Flynn, Kells, Joyce, Corcoran, Herley, Suarez, Cotter, Hurley, Weihrauch and Groeger^38^ found significant reduction in the sense of caregiver burden, as well as grief and depression, along with an increase in mastery, while the OTAU group did not show statistically significant improvements in any outcomes. Liljedahl, Kleindienst, Wangby‐Lundh, Lundh, Daukantaite, Fruzzetti and Westling^39^ evaluated the standard FC training (FC‐S; n = 34) compared to an intensified weekend training (FC‐R; n = 48) for the treatment of families of individuals receiving DBT. Both groups showed significant improvements in functioning, overall family function, and perceived resources in caring for their loved ones. Although these studies have focused on family members in general, there is a gap in evaluating the effectiveness of FC for family members of youth with MH challenges. Additional research by teams of investigators not involved in the original development of FC is needed to further examine its effectiveness and feasibility, as well as to consider its utility specifically for family members of youth with MH challenges.

### 1.3 Present study

Despite the high rates of MH challenges among youth and the significant challenges this poses on families, there is a dearth of less resource‐intensive treatment services designed to address the needs of family members. This may be largely due to the exclusion and discrimination family members have been found to experience when attempting to interact with health and mental health services.^16^ Family members of youth constitute an underserved population due to the social, emotional, and psychological challenges they experience as a result of caring for a youth with MH challenges. They are a population with a high need for support and services. FC is a promising program, as it can be facilitated by peers and could potentially have strong impact while being less resource‐intensive, beyond the domain of BPD. Thus, the present study examines the effectiveness and feasibility of FC as an intervention approach for families of youth with MH challenges. It was hypothesized that caregivers who participate in the 12‐week FC program would show reduced caregiver burden and parenting stress from pre‐treatment to follow‐up.

## 2 METHOD

### 2.1 Design and procedures

This study was part of the Research and Action for Teens (RAFT) project,^40^ a multi‐centre program of research that included an effectiveness evaluation of FC adapted for caregivers of youth with MH challenges. The Ontario‐based study used a mixed between and within subject pre‐post and follow‐up design to evaluate the change over time for caregivers of youth with MH challenges who were enrolled in a FC intervention in Toronto, Ottawa and Thunder Bay, Ontario, Canada. Participants completed all measures at baseline, mid‐skills training (6 weeks), post‐intervention (after 12‐week skills training) and at a 12‐week follow‐up. The FC intervention encompasses 12 weekly 90‐minute sessions in the form of a skills training group. Manualized modules focused on psychoeducation, individual skills and relationship skills to promote emotional well‐being, family skills to improve the quality of family relationships and interactions, effective self‐expression and problem management skills. Caregivers within the RAFT study were engaged as service providers.

### 2.2 Recruitment

Participants in the current study were recruited through flyers distributed to clinicians and posted on notice boards at the three participating treatment sites, as well as through relevant practitioner electronic mailing lists. Individuals interested in participating received a full description of the study and were screened by a research assistant to determine eligibility. Written informed consent was obtained from participants prior to enrolment. Remuneration was provided in the form of a $25 Gift Card for each assessment completed. The study was undertaken with approval by the Centre for Addiction and Mental Health Research Ethics Board, Royal Ottawa Health Care Group Research Ethics Board and the Children's Centre Thunder Bay Research Ethics Committee.

### 2.3 Participants

The sample included 94 participants from 3 cities: Toronto (N = 37), Thunder Bay (N = 22) and Ottawa (N = 35). Inclusion criteria were as follows: (a) 18 years of age or older, (b) literacy in English, (c) caregiver or family member of an adolescent between the ages of 14‐18, (d) reported adolescent behaviour in the clinical range on at least one subscale of the Child Behavior Checklist (CBCL), and (e) did not have an adolescent participating in the other arm of the RAFT study providing a youth intervention. Table 1 displays descriptive statistics on the demographic characteristics of the participants within and across the three sites.

**TABLE 1:** Participant characteristics reported at baseline, frequency (%) unless indicated otherwise

	Site				Group comparison*
	Ottawa	Thunder Bay	Toronto	Total	
n	35	22	37	94	
Age (mean [SD])	49.34 (5.50)	44.0 (7.76)	50.32 (7.83)	48.48 (7.40)	
Sex					
Male	11 (31.4%)	1 (4.5%)	12 (32.4%)	24 (25.5%)	*P* = .036
Female	24 (68.6%)	21 (95.5%)	25 (67.6%)	70 (74.5%)	
Caregiver ethnicity					
White	32 (91.4%)	21 (95.5%)	33 (89.2%)	33 (89.2%)	*P* = .053
Other	0 (0%)	0 (0%)	4 (10.8%)	4 (10.8%)	
Not reported	3 (8.6%)	1 (4.5%)	0 (0%)	4 (4.3%)	
Child's ethnicity					
White	33 (94.3%)	21 (95.5%)	31 (83.8%)	85 (90.4%)	*P* = .209
Other	2 (5.7%)	1 (4.5%)	6 (16%)	9 (9.6%)	
Caregiver's involvement with child					
Minimal involvement	3 (8.6%)	3 (13.6%)	5 (13.5%)	11 (11.7%)	*P* = .768
Very involved	32 (91.4%)	19 (86.4%)	32 (86.5%)	83 (88.3%)	
Current versus past involvement with child					
Less involved than in the past	7 (20.0%)	4 (18.2%)	12 (32.4%)	23 (24.5%)	*P* = .347
Same or more involved	28 (80.0%)	18 (81.8%)	25 (67.6%)	71 (75.5%)	
Household income					
Less than $10 000	0 (0.0%)	2 (9.1%)	0 (0.0%)	2 (2.1%)	*P* = .053
Between $10 000 and $39 999	1 (2.9%)	3 (13.6%)	4 (10.8%)	8 (8.5%)	
Over $40 000	34 (97.1%)	17 (77.3%)	33 (89.2%)	84 (89.4%)	
Employment status					
Working	30 (85.7%)	16 (72.7%)	32 (86.5%)	78 (83.0%)	*P* = .342
Not working	5 (14.3%)	6 (27.3%)	5 (13.5%)	16 (17.0%)	
Number of family members in household					
1 to 2	5 (14.3%)	1 (4.5%)	11 (29.7%)	17 (18.1%)	*P* = .057
3 to 4	23 (65.7%)	12 (54.5%)	19 (51.4%)	54 (57.4%)	
5+	7 (20.0%)	9 (40.9%)	7 (18.9%)	23 (24.5%)	
Education level					
High school diploma or higher	27 (77.1%)	14 (63.6%)	30 (81.1%)	71 (75.5%)	*P* = .309
No high school diploma	8 (22.9%)	8 (36.4%)	7 (18.9%)	23 (24.5%)	
Marital status					
Married	29 (82.9%)	16 (72.7%)	26 (70.3%)	71 (75.5%)	*P* = .435
Not married	6 (17.1%)	6 (27.3%)	11 (29.7%)	23 (24.5%)	

* Chi‐square p‐value compares site and participant characteristics at baseline

### 2.4 Treatment and therapists

The 12‐week FC intervention was adapted for family members of youth with MH challenges in consultation with the treatment developers. Although the FC intervention is generally delivered by family members to family members, in this adaptation, a combination of service providers and family members delivered the services due to existing institutional requirements. In Toronto, groups were co‐led by a clinician and a family member in a tertiary care centre; in Thunder Bay, groups were led by clinicians only in a community‐based child and youth mental health agency; in Ottawa, groups were led by family members only in a community‐based peer‐run organization. All facilitators completed a full FC training workshop led by one of the treatment developers and were provided consultation by experienced DBT therapists, who received intensive training in the FC intervention. Bi‐weekly consultation was provided by a senior therapist.

### 2.5 Measures

#### 2.5.1 Caregiver burden

The Burden Assessment Scale (BAS) is a 19‐item measure examining both objective and subjective consequences of providing ongoing care to individuals with serious mental health challenges. Ten items assess burden resulting from caregiving responsibilities by examining financial distress, limitations to engaging in personal activities, disruptions to the household routines and negative effects on social interactions. The remaining nine items measure aspects of subjective burden, including the feelings, attitudes and emotions encompassing the caregiver experience including shame, stigma, guilt and worry.^41^ The BAS shows good psychometric properties including internal consistency (Cronbach's α = 0.91).^41^

#### 2.5.2 Caregiver stress

The Stress Index for Parents of Adolescents (SIPA)^42^ is an adolescent version of the Parenting Stress Index.^42^ The SIPA was developed to assess stress within parent‐adolescent interactions from the perspective of parents. Responses to the 112 items yield scores in a number of domains including stress, including an Index of Parenting Stress, and Adolescent Stress Domain, and a Parent Domain, representing the adolescent's behaviour, parenting skills and quality of the parent‐adolescent relationship. The SIPA has sound psychometric properties including internal consistency (Cronbach's α = 0.88).^43^

#### 2.5.3 Caregiver report of child's behaviour

The Child Behaviour Checklist (CBCL)^44^ is a measure within the Achenbach System of Empirically Based Assessment (ASEBA) that is used to examine parents’ perspectives of their child's behaviour and emotional problems. The CBCL is made up of 113 items to measure internalizing (e.g. anxious) and externalizing (e.g. aggressive behaviour) problem behaviours. Evidence for content, construct and criterion‐related validity is well documented. Internal consistency and test‐retest reliability are strong for the CBCL scales (Cronbach's α ranged from α = 0.75 to α = 0.84), with test‐retest coefficients ranging from 0.78 to 0.88.^45^

#### 2.5.4 Affect

The Family Experience Interview Schedule (FEIS)^46^ was designed and tested for use with family members of individuals with severe mental illness. It is typically administered through a personal interview format. A multidimensional approach to the family experience facilitates distinguishing between different aspects of caregiver burden and related constructs. The following dimensions of caregiver burden are measured: assistance in daily living, supervision of bothersome or troublesome behaviours, impact on daily routine, financial expenditures and affective responses.^46^ The FEIS has demonstrated sound psychometric properties including internal consistency (Cronbach's α = 0.92) and criterion‐related validity.^46^

#### 2.5.5 Mastery

The Pearlin Mastery Scale (MS)^47^ is a 7‐item questionnaire used to measure the extent to which an individual regards their life chances as being under their control as opposed to being ruled by fate.^47^ The MS has been shown to have good construct and predictive validity and good internal consistency according to classical test theory criteria (Cronbach's α = 0.78).^48^, ^49^

#### 2.5.6 Coping

The DBT‐Ways of Coping Checklist (DBT‐WCCL)^50^ was adapted from the Revised Ways of Coping Checklist (RWCCL). It measures the use of DBT‐based coping skills and dysfunctional coping skills within the last month and helps clinicians determine if caregivers are utilizing the DBT skills taught. DBT‐WCCL is comprised of 59 items examining the frequency of DBT skill use in the last month and the frequency of non‐DBT dysfunctional coping strategies.^51^ Internal consistency, test‐retest reliability and content validity analyses suggest that the scale has good to excellent psychometric properties (Cronbach's α for subscales ranged from α = 0.84 to α = 0.96) ^50^

#### 2.5.7 Grief

The Grief Scale^52^ is a 15‐item measure of current feelings of grief associated with the mental illness of a loved one. Higher scores represent more intense experiences of grief. The GS shows strong internal consistency (Cronbach's α = 0.92).^52^

### 2.6 Analyses

The frequency distributions of participant's characteristics at baseline were reported, with chi‐square tests for comparison across sites. The effects of the intervention on the primary and secondary outcome measures were assessed using a linear mixed effect model to estimate change over time. The outcome measures were defined as the dependent variable. Time (baseline, 6 weeks, 12 weeks and follow‐up), site (Toronto, Thunder Bay and Ottawa) and the time × site interaction were defined as the fixed effects. All analyses were adjusted for age. Type III tests of fixed effects were used to determine the main effects among the dependent variables for time and time × site. Estimates of Fixed Effects were used to determine significance among the coefficient (ß) of each variable, where the time reference was defined as the last time point (follow‐up). Lastly, estimated marginal means were examined for time and time × site. The analyses for both primary and secondary outcomes were performed using the IBM SPSS statistics software (Version 25).^53^

#### 2.6.1 Primary outcomes

The primary outcomes of this study were the determination of estimated mean changes for BAS and SIPA between baseline and follow‐up, assessed via a linear mixed effect model to estimate change over time and across sites. The mixed effect model was fitted such that individual subjects were defined as random effects.

#### 2.6.2 Secondary outcomes

The secondary outcomes include the estimated mean changes of the following scales, between baseline and follow‐up: CBCL, FEIS, Mastery Scale, DBT‐WCCL and Grief Scale. The secondary analyses were performed using a linear mixed‐effects model for these measures. Results from subscales of these measures are reported when considered clinically relevant. In the model used for the secondary outcomes, the individual subjects were defined as random effects.

## 3 RESULTS

### 3.1 Demographics

The demographic characteristics are shown in Table 1. Of the 94 participants included in this study (n = 35 for Ottawa, n = 22 for Thunder Bay, n = 37 for Toronto), data were available for 75 (80%) at six weeks, 65 (69%) at 12 weeks and 63 (67%) at follow‐up. The mean age of participants across the sites was 48.48 (SD = 7.40), and 74.5% of participants were female. The majority of participants identified as White (89.2%), had high school as their highest educational level (75%) and were married (64%). Most demographics were equivalent across sites, with the exceptions of a statistically significant difference for sex (*P* = .036).

### 3.2 Primary outcomes

**FIGURE 1:**
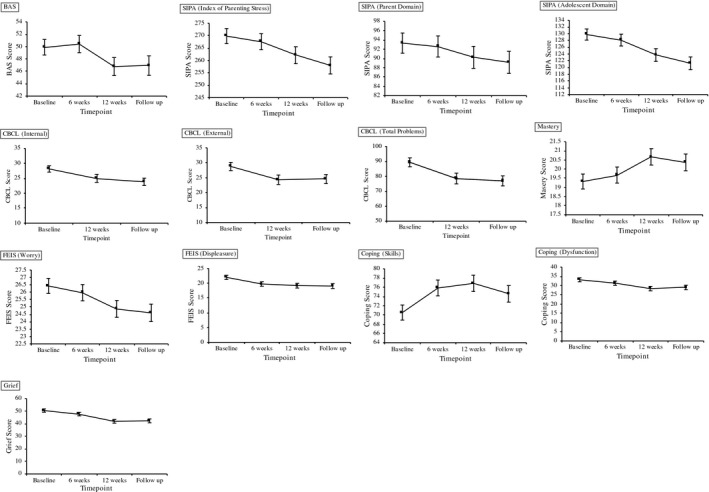
The estimated marginal means of primary and secondary outcome variables for time. Estimated marginal means with standard error for each timepoint are shown for: Burden Assessment Scale (BAS), Stress Index for Parents of Adolescents (SIPA), Child Behaviour Checklist (CBCL), Family Experience Interview Schedule (FEIS), Mastery Scale, DBT‐Ways of Coping Checklist DBT‐WCCL) and Grief Scale

The estimate marginal means (EMM) for primary and secondary outcomes variables for the predictor variable time are reported in Table 2 and Figure 1. The coefficient estimates (ß) with respect to time and the time × site interaction are presented in Tables 3, in which the time reference for all scales was defined as follow‐up. The type III analysis of fixed effects for the time, site, age and time × site variables are provided as supplemental materials.

**TABLE 2:** Estimate Marginal Means (SE) for primary and secondary outcomes variables from baseline to follow‐up

	Estimate Marginal Means (SE) for Time				F	*P* ^a^
	Baseline	6 wk	12 wk	Follow‐up		
Primary outcome variables						
BAS	49.90 (1.26)	50.41 (1.39)	46.77 (1.46)	46.93 (1.58)	3.376	.019
SIPA (Index of Parenting Stress)	269.84 (3.05)	267.57 (3.24)	262.13 (3.42)	258.00 (3.44)	7.251	<.001
SIPA (Adolescent Domain)	129.78 (1.65)	128.14 (1.76)	123.71 (1.88)	121.25 (1.89)	11.555	<.001
SIPA (Parent Domain)	93.310 (2.17)	92.59 (2.27)	90.24 (2.37)	89.16 (2.38)	2.551	.057
Secondary outcome variables						
CBCL (Internal Problems)^b^	28.16 (1.07)	‐	25.01 (1.34)	23.84 (1.23)	8.076	.001
CBCL (External Problems)^b^	28.75 (1.37)	‐	28.75 (1.58)	24.53 (1.51)	10.281	<.001
CBCL (Total Problems)^b^	89.41 (2.98)	‐	78.56 (3.60)	76.99 (3.36)	11.520	<.001
FEIS (Worry Scale)	26.42 (0.51)	25.96 (0.55)	24.87 (0.57)	24.60 (0.59)	5.029	.002
FEIS (Displeasure Scale)	21.998 (0.70)	19.75 (0.76)	19.17 (0.79)	19.05 (0.82)	6.578	<.001
Mastery	19.322 (0.41)	19.67 (0.44)	20.67 (0.45)	20.35 (0.46)	5.009	.002
DBT‐WCCL (Skills)	70.527 (1.63)	75.86 (1.72)	76.85 (1.76)	74.58 (1.80)	10.282	<.001
DBT‐WCCL (Dysfunction)	33.109 (1.08)	31.27 (1.15)	28.34 (1.19)	28.95 (1.21)	11.366	<.001
Grief Scale	50.438 (1.32)	47.65 (1.42)	41.86 (1.46)	42.25 (1.54)	20.184	<.001

Note

*P*‐values are reported for the respective dependent variable interaction with time through type III tests of fixed effects.

^a^ Indicated for significant interaction effect at α = 0.05.

^b^ Data were not collected for CBCL in the 6 weeks timepoint.

**TABLE 3:** Coefficient estimates with respect to time, for primary and secondary outcome measures and coefficient estimates with respect to the time by site interaction, for primary outcome measures

Variable	Outcome ß (95% CI) Reference	*P*
Primary outcome variables		
BAS		
Time^a^	7.33 (3.58, 11.08)	<.001
Time × Site (Ottawa)^a^	−1.96 (−7.76, 3.84)	.51
Time × Site (Thunder Bay)^a^	−11.12 (−18.90, −3.34)	.005
SIPA (Index of Parenting Stress)		
Time^a^	16.19 (8.95, 23.43)	<.001
Time × Site (Ottawa)ª	−0.22 (−11,36, 10.91)	.97
Time × Site (Thunder Bay)^a^	−12.80 (26.83, 1.23)	.07
SIPA (Adolescent Domain)		
Time^a^	9.58 (5.35, 13.81)	<.001
Time × Site (Ottawa)ª	2.49 (−4.00, 8.99)	.45
Time × Site (Thunder Bay)^a^	−5.67 (−13.84, 2.51)	.17
SIPA (Parent Domain)		
Time^a^	8.13 (3.68, 12.57)	<.001
Time × Site (Ottawa)^a^	−3.59 (−10.43, 3.26)	.30
Time × Site (Thunder Bay)^a^	−8.36 (−16.99, 0.27)	.06
Secondary outcome variables		
CBCL (Internal Problems)		
Time^a^	6.35 (3.29, 9.41)	<.001
CBCL (External Problems)		
Time^a^	5.53 (2.56, 8.49)	<.001
CBCL (Total Problems)		
Time^a^	17.74 (10.05, 25.44)	<.001
FEIS (Worry Scale)		
Time^a^	2.36 (0.94, 3.77)	.001
FEIS (Displeasure Scale)		
Time^a^	4.15 (2.12, 6.19)	<.001
Mastery		
Time^a^	−0.66 (−1.70, 0.38)	.21
DBT‐WCCL (Skills)		
Time^a^	−5.26 (−8.72, −1.80)	.003
DBT‐WCCL (Dysfunction)		
Time^a^	4.12 (1.66, 6.59)	.001
Grief Scale		
Time^a^	8.71 (5.23, 12.18)	<.001

Note

Model is adjusted by age.

^a^ Baseline to follow‐up, such that follow‐up is the reference value (ß = 0)

#### 3.2.1 Caregiver burden

**FIGURE 2:**
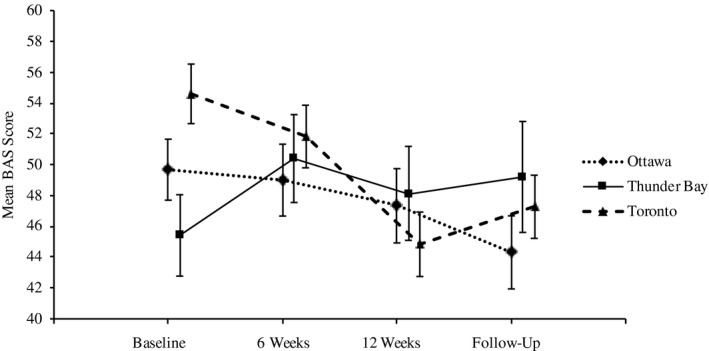
The estimated marginal means of the BAS for time by site interaction. The Burden Assessment Scale (BAS) estimated marginal means with standard error for the time by site interaction are shown for Ottawa, Toronto and Thunder Bay.

The linear mixed level analysis showed a statistically significant improvement in the BAS for the predictor variable time (ß = 7.33, 95% CI: [3.58, 11.08]; *P* < .001). The time × site interaction was also significant (ß=−11.12, 95% CI: [−18.90, −3.34]; *P* = .005) for Thunder Bay, with Toronto as the reference value, showing that the time effect may not have been observed in the same manner in Thunder Bay as compared to Toronto. The EMM of the BAS for the time × site interaction is shown in Figure 2.

#### 3.2.2 Stress

SIPA scores showed a statistically significant reduction in perceived stress between baseline and follow‐up in the Index of Parenting Stress (ß = 16.19, 95% CI: [8.95, 23.43]; *P* < .001) and in the Adolescent Domain Stress (ß = 9.58, 95% CI: [5.35, 13.81]; *P* < .001). The EMMs for SIPA between baseline and follow‐up are displayed in Table 2. The type III analysis showed no statistically significant changes in the Parent Domain over time (*P* = .057). The time × site interaction for SIPA was not significant. See Tables 3 for SIPA results.

### 3.3 Secondary outcomes

#### 3.3.1 Child behaviour

Participants demonstrated a statistically significant reduction in scores on the CBCL from baseline to follow‐up for Internal Problems (ß = 6.35, 95% CI: [3.29, 9.41]; *P* < .001) and External Problems ß = 5.53, 95% CI: [2.56, 8.49]; *P* < .001). The CBCL EMMs between baseline and follow‐up are shown in Table 2. There was no significant difference in CBCL results for the time × site interaction (Internal Problems: *P *= .495: External Problems: *P *= .086). See Tables 3 for CBCL results.

#### 3.3.2 Affect

The Worry Scale of the FEIS revealed statistically significant improvements between baseline and follow‐up (ß = 2.36, 95% CI: [0.94, 3.77]; *P* = .001). Ratings on the FEIS Displeasure Scale revealed a statistically significant decrease in perceptions of displeasure from baseline to follow‐up (ß = 2.36, 95% CI: [0.94, 3.77]; *P* < .001). The EMMs for the FEIS between baseline and follow‐up are reported in Table 2. Scores for the FEIS did not show a statistically significant improvement in the time × site interaction (Worry Scale: *P* = .273; Displeasure Scale: *P* = .272). See Tables 3 for FEIS results.

#### 3.3.3 Mastery

The change in scores on the Mastery Scale between baseline and follow‐up was statistically significant (ß=−0.66, 95% CI: [−1.70, 0.38]; *P* = .002 for type III analysis of fixed effects). The EMMs for the Mastery Scale between baseline and follow‐up are reported in Table 2. There was no statistically significant time × site interaction (*P* = .761). See Tables 3 for Mastery Scale results.

#### 3.3.4 Coping

A statistically significant improvement in the use of DBT skills from baseline to follow‐up was observed (ß = −5.26, 95% CI: [−8.72, −1.80]; *P* = .003). Significant decreases in dysfunctional coping were also revealed (ß = 4.12, 95% CI: [1.66, 6.59]; *P* = .001). The EMMs for the DBT‐WCCL Scale between baseline and follow‐up are reported in Table 2. Changes in the use of coping skills and ratings of dysfunction were not statistically significant for the time × site interaction (Coping Skills: *P = *.726; Dysfunction: *P* = .556). See Tables 3 for DBT‐WCCL results.

#### 3.3.5 Grief

A statistically significant improvement on the Grief Scale between baseline and follow‐up was observed (ß = 8.71, 95% CI: [5.23, 12.18]; *P* < .001). The EMMs for the DBT‐WCCL Scale between baseline and follow‐up are reported in Table 2. There was no statistically significant time × site interaction for the Grief Scale (*P* = .660). See Tables 3 for Grief Scale results.

## 4 DISCUSSION

This study investigates the impact of a 12‐week FC intervention adapted for caregivers of youth with MH challenges. The results indicate favourable responses to the intervention. The primary outcomes, that is caregiver burden and parenting stress, improved over time, with one interaction effect showing less impact on caregiver burden in the Thunder Bay site. Improvements were also observed in most secondary outcome measures, including child behavioural concerns, affect, mastery and coping, across all sites. All outcome measures, aside from the SIPA (Parent Domain) showed statistically significant improvements over time. These findings are consistent with previous studies evaluating the effects of FC in the United States^36^, ^54^ and in Europe.^37^, ^38^ As previous studies are family member focused rather than youth focused, this suggests an adaptation of FC for caregivers of youth with MH challenges may be a potentially beneficial intervention for this population.

In previous studies, participants involved in the FC program demonstrated reduced grief and burden, mirroring our findings of significant decreases in burden and grief between baseline and follow‐up.^36^, ^37^, ^38^, ^39^ Grief has been previous highlighted as a contributing factor to the burden and support needs of families and caregivers of persons with complex mental health challenges.^55^ Findings from this study suggest that FC is effective in alleviating the sense of grief among participants.

In this study, different treatment facilitation models were employed. In Toronto, a peer facilitator led the group alongside a clinician, while in Ottawa the group was led exclusively by peer facilitators. In contrast, the Thunder Bay site adopted a clinician‐led model. Since caregiver burden did not change over time for the Thunder Bay site, it is possible that peer facilitation is one of the key elements supporting the effectiveness of FC. Since the literature suggests the benefits of peer facilitation for family members, such as emotional support, feelings of acceptance, increased care giving satisfaction, empowerment, increased coping skills and an increase in program attendance,^20^, ^21^, ^22^ peer facilitation may be a key mechanism of action in the impact of FC.

Previous FC studies are limited in the considerations of geographical site differences with the exception of the Liljedahl, Kleindienst, Wangby‐Lundh, Lundh, Daukantaite, Fruzzetti and Westling^39^ study, which looked at FC in two different therapeutic intensities among geographically isolated families. Thus, this study is one of the first to include three different geographical locations in the study design. Another possible explanation for differential site for caregiver burden is the geographical differences between the three sites. In comparison to the other sites, Thunder Bay has the smallest and most northern population of the three sites. Accordingly, it may be that Northern populations may require additional tailoring of service delivery practices to reflect the unique needs of this population. Researchers have discussed the geographical limitations of services in northwestern Ontario, where adequate mental health services appear to be less accessible.^56^ Therefore, geographical location, combined with local contextual and demographic features, may have affected the outcome in this location. Nevertheless, the remaining outcome measures did show significant improvements in the Thunder Bay location, suggesting some level of effectiveness for FC with different models and in different locations. Potential differences among sites and facilitation models should be further explored in future evaluations of FC.

### 4.1 Strengths and Limitations

The study contributes to the literature by reporting on the outcomes of 94 caregivers participating in FC, specifically adapted for caregivers of youth with MH challenges. It included three sites with varying geographical locations and facilitation models. While the results of this study are promising, there are some limitations to keep in mind. Firstly, there was no control group, and therefore, it is not possible to determine whether the changes observed were due to participating in the program or another factor (e.g., passage of time). Secondly, it was not possible to determine whether the differential site effects were due to the facilitation model, geographical location, demographic features or other site‐specific variables. Thirdly, the participant demographics were limited with regards to ethnicity and other social determinants that may influence well‐being. The sample may not have been representative of a general population of caregivers of youth with MH challenges, and findings may not be generalizable. Lastly, there were no measures provided by other family members, such as youth. Further research is needed to tease apart these effects and to determine the specific mechanisms of change within FC that are most influential in improving outcomes.

## 5 CONCLUSION

This study adds to the literature by demonstrating the effectiveness of FC as an intervention for caregivers of youth with MH challenges. FC appears to offer caregivers of youth with MH challenges an opportunity to acquire skills and improve coping with complex emotions in a supportive group setting, while reducing overall burden and stress. Further research is needed to determine the differences of FC across geographical sites and facilitation models, while examining the barriers and facilitators to flexible implementation in varying settings and with varying participant profiles.

## CONFLICT OF INTEREST

The authors declare no conflicts of interest.

## AUTHORS’ CONTRIBUTIONS

Tali Boritz (joint first author) contributed to the conception, design, acquisition of data, revising manuscript and final approval of version to be published. Natasha Y. Sheikhan (joint first author) contributed to the analysis, interpretation of data, and drafting and revising the manuscript. Lisa Hawke contributed to the analysis, interpretation of data, revising manuscript, revising manuscript and final approval of the version to be published. Shelley McMain contributed to the conception, design, acquisition of data and revising the manuscript and final approval of the version to be published. Joanna Henderson (senior author) contributed to the conception, design, revising of the manuscript and the final approval of version to be published.

## Data Availability

The data that support the findings of this study are available from the corresponding author upon reasonable request.
